# Safety of Ligation of Aberrant Left Hepatic Artery Originating from Left Gastric Artery in Laparoscopic Gastrectomy for Gastric Cancer

**DOI:** 10.1038/s41598-020-62587-7

**Published:** 2020-04-03

**Authors:** Rene Ronson G. Ang, Hyuk-Joon Lee, Jae Seok Bae, Chun-Chao Zhu, Felix Berlth, Tae Han Kim, Shin-Hoo Park, Yun-Suhk Suh, Seong-Ho Kong, Se Hyung Kim, Han-Kwang Yang

**Affiliations:** 1 Department of Surgery, Seoul National University Hospital, Seoul National University College of Medicine, Seoul, Korea; 2 Cancer Research Institute, Seoul National University College of Medicine, Seoul, Korea; 3 Department of Radiology, Seoul National University Hospital, Seoul, Korea; 4 Department of Gastrointestinal Surgery, Renji Hospital, Shanghai Jiaotong University School of Medicine, Shanghai, China; 5 Department of General, Visceral and Cancer Surgery, University of Cologne, Köln, Germany; 6 Department of Surgery, Gyeongsang National University Changwon Hospital, Changwon, Korea; 7 Department of Surgery, Cebu Doctors’ University Hospital, Cebu City, Philippines; 8 Department of Surgery, University of Cebu Medical Center, Cebu City, Philippines

**Keywords:** Gastric cancer, Surgical oncology

## Abstract

There are still lot of controversies whether aberrant left hepatic artery (ALHA) originating from left gastric artery should be ligated or preserved during gastric cancer (GC) surgery. We aimed to investigate this issue. We reviewed ALHA cases who had laparoscopic gastrectomy for gastric cancer at Seoul National University Hospital (SNUH) from 2012 to 2016. Type of ALHA variants using Michel’s classification of hepatic arterial anatomy and diameter of each vessel were evaluated by 2 radiologists. Postoperative hepatic function and surgical outcome were collected until 6 months after surgery. Results showed that if the diameter of ALHA was larger than 1.5 mm, a transient elevation of SGOT and SGPT on postoperative day 2 was observed in the ligated cases. No differences were observed in operation time, amount of blood loss, overall complication rate, hospital stay, and number of lymph nodes retrieved between the ligated and preserved replaced left hepatic artery (RLHA) and accessory left hepatic artery (acLHA) group. In this study, we conclude that ligation of ALHA seems to be safe as none of the patients suffered adverse outcome. A transient rise in postoperative SGOT and SGPT levels were seen after ligating ALHA >1.5 mm in diameter regardless of subtype.

## Introduction

About 25–75% of the population have variation in hepatic artery anatomy^1^. Twenty five percent of these are aberrant left hepatic artery (ALHA) originating from left gastric artery^2^ of which 55% and 45% are replaced (RLHA) and accessory (acLHA) type^3^. Recent data showed that the worldwide incidence of ALHA is 13.52% and for replaced and accessory type, 8.26% and 5.55% respectively^4^. These hepatic artery variants are occasionally seen during gastric cancer surgery and preservation of these arteries with complete removal of surrounding lymph nodes especially in laparoscopic surgery is challenging. Ligation or preservation of these arteries is of great concern to surgeons regarding patient’s postoperative liver function, safety and oncologic outcome.

Oncologic gastric surgery requires complete station 7 lymph node dissection^5^. In order to achieve this, left gastric artery should be ligated at the base to ensure en bloc lymph node dissection which means that when ALHA is present it should be ligated. Many literatures recommend preservation of ALHA during surgery especially in patients with pre-existing liver disease to prevent liver dysfunction and ischemia^6,7^. Although there were no difference in oncologic outcome between patients whose aberrant left hepatic artery were preserved or ligated during gastric cancer surgery as reported by Shinohara *et al*.^8^, many surgeons believed that preserving ALHA during surgery increases the operation time and blood loss especially during laparoscopic procedures. Mays *et al*.^9^ and Lee *et al*.^10^ have demonstrated in their studies that revascularization thru collateral flow takes place after ligation of hepatic arterial inflow^8^ and there were case reports of ligation of ALHA^11^, hepatic artery proper^12^ for ruptured aneurysm and accidental ligation of hepatic artery^13^ which showed no morbidity and mortality. The objective of this study is to determine if ligation of ALHA during laparoscopic gastrectomy for gastric cancer is safe with regards to patient’s postoperative short-term outcome and oncologic safety.

## Methods

### Patients

Medical records of 2,487 patients who had laparoscopic gastrectomy for gastric cancer at Seoul National University Hospital from January 2012 and December 2016 were reviewed. The presence of ALHA were initially noted on multidetector computed tomography (MDCT) reports. To confirm presence of ALHA and to check whether it was preserved or ligated, full-version video of each operation was reviewed.

Two radiologists independently reviewed each patient’s CT scan and categorized ALHA into either RLHA (Type II or IV) or acLHA (Type V, VII or VIII) based on Michel’s classification of hepatic arterial anatomy. The diameter of ALHA were also measured. Patients were then categorized into four groups: RLHA-ligated and preserved group and acLHA-ligated and preserved group. Staging of the gastric cancer was based on the International Union Against Cancer [UICC] TNM Classification, 7th edition. Patients who had palliative gastric surgery, history of previous gastric or liver surgery and combined gastric and liver operations were excluded from the study. This study was approved by the SNUH Institutional Review Board (IRB) with IRB no. H-1705-018-851. All procedures done were in accordance with the ethical standards. Because the risk is minimal for the patient, informed consent was waived by the Institutional Review Board.

### Imaging

MDCT scanners with 16–256 detector rows (Definition, Siemens medical system; Brilliance 16, Philips Healthcare; Discovery HD750, GE healthcare) were used in the preoperative imaging. Stomach protocol CT consists of two phases for early gastric cancer (EGC) and three phases for advanced gastric cancer (AGC). For EGC, arterial and portal phase CT scan were obtained in right anterior oblique position. A delayed phase obtained in right down decubitus position was added for AGC. The following are CT acquisition parameters for MDCT: tube voltage of 100–120 kVp; tube current of 150–250 mAs; slice thickness of 1.3–3 mm; reconstruction interval of 0.7–3 mm; pitch of 0.9–1; rotation time of 0.5–1 sec. For contrast administration, an automatic power injector was used with iodinated contrast agent (350 or 370 mg·I/ml) at a rate of 3–5 ml/sec and at a dose of 1.5–1.6 ml/kg for 30 seconds. Saline chase was performed at the same rate for 10 seconds. By using the bolus tracking method, arterial phase scan was started 18–23 sec after the enhancement threshold (100 Hounsfield Unit HU) was reached in the descending thoracic aorta. For portal venous phase scan, a fixed delay of 65–75 sec was used.

### Surgical procedure

The type of gastric resection (i.e. distal gastrectomy, proximal gastrectomy, pylorus-preserving gastrectomy or total gastrectomy) and the extent of lymphadenectomy (D1, D1 + or D2) were based on the TNM stage and location of the tumor as stated in the Japanese Gastric Cancer Association treatment guidelines 2014 (ver. 4)^14^. During surgery, tissues and lymph nodes located in the vicinity of the common hepatic (station 8), celiac axis (station 9) and proximal splenic artery (station 11p) were dissected to expose the origin of the left gastric artery. If an ALHA was encountered during dissection of the gastrohepatic ligament, if it were to be preserved, fat and lymphoid tissues around the entire course of the vessel were skeletonized during dissection and then ligated distal to the branching from the LGA. If ALHA were to be sacrificed, LGA was ligated at root of the vessel.

### Postoperative evaluation

Postoperative short term outcome such as operation time, amount of blood loss, length of hospital stay after surgery and number of harvested lymph nodes were compared between each groups. Amount of blood loss was calculated based on the operative records. Liver function tests such as SGOT, SGPT, alkaline phosphatase and total bilirubin and surgical morbidity were reviewed at postoperative day 2, day 5, 2 weeks, 3 and 6 months using Clavien-Dindo classification of surgical complications^15^.

### Statistical analysis

Data were analyzed using SPSS 20.0 (SPSS Inc, Chicago, IL, USA) and GraphPad Prism 7 (San Diego, CA) software. Chi-square test was used to compare patient characteristics and surgical complications. Liver function test results, operation outcome and number of harvested lymph nodes in each station on the lesser curvature side of the 4 groups were compared using Student t test. Pearson correlation and linear regression were used to correlate liver function parameter (SGOT, SGPT) with diameter of ALHA in ligated patients. Less than 0.05 p-value was considered statistically significant.

## Results

Of the 2,487 laparoscopic gastrectomy cases screened, ALHA was seen in 442 (17.7%) patients of which 204 (8.2%) patients with complete data including operation video were included in the study. In the 204 patients, 131 (64.2%) were classified as replaced and 73 (35.8%) as accessory left hepatic artery and these arteries were preserved in the 135 (66.2%) cases and ligated in 69 (33.8%) cases.

### Patient’s demographics

Patient characteristics are presented in Table 1. There are 204 patients included in the study, 140 are men and 64 are women. No significant differences were seen among the 4 groups in terms of age, gender, body mass index (BMI), surgery type and T and N stage (P > 0.05). There were 21 patients with pre-existing hepatic disease which includes drug induced liver injury (DILI), hepatitis infection and liver cirrhosis, 14 of these patients are in the RLHA-preserved group, 1 in acLHA-preserved, 3 in RLHA-ligated and 3 in acLHA-ligated group.

**Table 1:** Patient demographics.

Variables	Replaced LHA (n = 131)			Accessory LHA (n = 73)		
	Preserved n = 114,(%)	Ligated n = 17,(%)	*P* value	Preserved n = 21,(%)	Ligated n = 52,(%)	*P* value
**Age (yrs)**			0.116			0.796
≥60	62 (54.4)	13 (76.5)		14 (66.7)	33 (63.5)	
<60	52 (45.6)	4 (23.5)		7 (33.3)	19 (36.5)	
**Sex**			0.265			0.132
Male	75 (65.8)	14 (82.4)		12 (57.1)	39 (75.0)	
Female	39 (34.2)	3 (17.6)		9 (42.9)	13 (25.0)	
**BMI (kg/m**^**2**^**)**			0.398			1.000
≥24	33 (28.9)	3 (17.6)		4 (19.0)	12 (23.0)	
<24	81 (71.1)	14 (82.4)		17 (81.0)	40 (77.0)	
**Diameter of ALHA (mm)**		0.413			0.411	
≥1.5	102 (89.5)	14 (82.4)		16 (76.2)	33 (63.5)	
<1.5	12 (10.5)	3 (17.6)		5 (23.8)	19 (36.5)	
**Surgery Type**			0.846			0.499
LDG	65 (57.0)	9 (52.9)		12 (57.1)	33 (63.5)	
LTG	13 (11.4)	3 (17.6)		2 (9.5)	7 (13.5)	
LPPG	34 (29.8)	5 (29.4)		7 (33.3)	10 (19.2)	
LPG	2 (1.8)	0 (0.0)		0 (0.0)	2 (3.8)	
**T stage**			0.736			0.185
T1	94 (82.5)	15 (88.2)		16 (76.2)	46 (88.5)	
T2–4	20 (17.5)	2 (11.8)		5 (23.8)	6 (11.5)	
**Lymph Node Metastasis**			0.392			0.216
No	103 (90.4)	14 (82.4)		17 (81.0)	48 (92.3)	
Yes	11 (9.6)	3 (17.6)		4 (19.0)	4 (7.7)	
**Pre-existing Liver Disease**			0.463			0.089
No	100 (87.7)	14 (82.4)		20 (95.2)	49 (94.2)	
Yes	14 (12.3)	3 (17.6)		1 (4.8)	3 (5.8)	

LHA = Left Hepatic Artery, LDG = Laparoscopic Distal Gastrectomy, LTG = Laparoscopic Total. Gastrectomy, LPPG = Laparoscopic Pylorus Preserving Gastrectomy, LPG = Laparoscopic Proximal Gastrectomy.

### Changes in liver function

**Figure 1:**
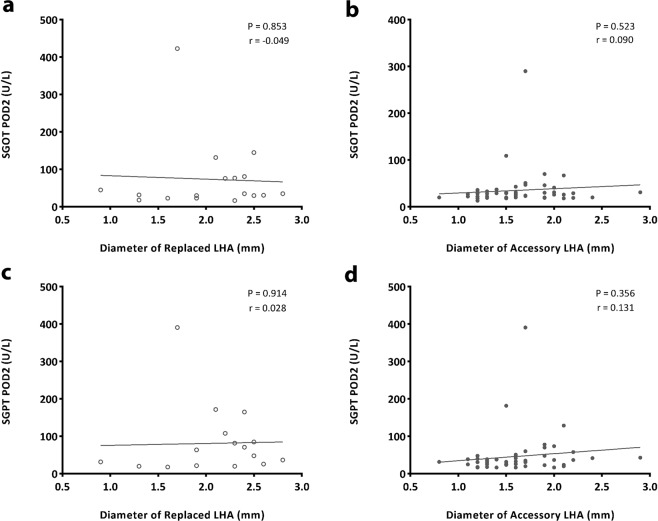
Correlation of liver function parameter (SGOT, SGPT) to diameter of aberrant LHA in ligated patients. There was no significant trend towards SGOT and SGPT elevation in either RLHA or acLHA (p = 0.853, 0.914, 0.523, 0.356 respectively) using the diameter of the vessel as continuous variable.

**Figure 2:**
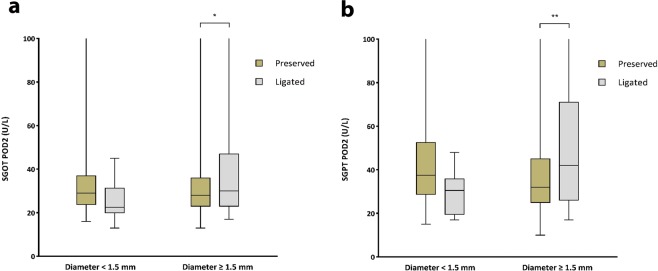
Comparison of SGOT/SGPT level at postoperative day 2 between <1.5 mm and ≥1.5 mm group regardless of subtype. A significant higher level of SGOT and SGPT were seen in the >1.5 mm group (*p = 0.010, **p = 0.001 respectively).

We reviewed the liver function tests done within 6 months postoperatively. Liver enzyme values are presented in Table 2. Significant higher levels of SGOT was seen on POD 2 in ligated RLHA group (p < 0.001) and significant higher levels of SGPT was seen on POD 2 and POD 5 in ligated RLHA group (p < 0.001, p 0.046). All values recovered completely two weeks after surgery without any medication or intervention. Mean total bilirubin level was slightly elevated at postoperative 6 months for the ligated RLHA group but we noted that there was no elevation of total bilirubin levels in the patient group except for one with Child’s B liver cirrhosis who had elevated baseline TB level preoperatively and on day 2,5, 2 weeks, 3 and 6 months postoperatively. We attributed this elevated TB level as a result of progression of the disease process. Differences in mean serum alkaline phosphatase were not significant between the 4 groups. The correlation of SGOT and SGPT levels with the diameter of the aberrant LHA in ligated patients were analyzed. There was no significant trend towards SGOT and SGPT elevation in either RLHA or acLHA (p = 0.853, 0.914, 0.523, 0.356 respectively) using the diameter of the vessel as continuous variable (Fig. 1) but when combining all ALHA ligated cases and divided into >1.5 mm and <1.5 mm diameter group, a significant higher level of SGOT and SGPT were seen on POD2 in the >1.5 mm group as shown in Fig. 2.

**Table 2:** Perioperative liver function tests of aberrant LHA patients.

Index	Replaced LHA (n = 131)			Accessory LHA (n = 73)		
	Preserved (n = 114)	Ligated (n = 17)	*P* value	Preserved (n = 21)	Ligated (n = 52)	*P* value
**SGOT (U/L)**						
Pre-op	24.1 ± 13.6	22.1 ± 9.6	0.559	20.9 ± 6.3	22.9 ± 13.4	0.508
Post-op (Day 2)	24.3 ± 21.8	73.7 ± 97.9	**<0.001**	34.7 ± 17.6	35.2 ± 39.4	0.952
Post-op (Day 5)	22.1 ± 8.7	25.4 ± 14.9	0.204	23.1 ± 10.8	23.2 ± 13.9	0.984
Post-op (2 weeks)	24.6 ± 11.2	30.4 ± 24.3	0.102	22.1 ± 7.3	21.9 ± 6.8	0.920
Post-op (3 months)	25.7 ± 11.7	29.8 ± 18.3	0.213	27.2 ± 14.4	24.3 ± 7.9	0.275
Post-op (6 months)	25.7 ± 10.0	24.8 ± 10.2	0.717	28.8 ± 13.3	25.0 ± 7.5	0.138
**SGPT (U/L)**						
Pre-op	22.4 ± 15.6	22.0 ± 10.0	0.918	18.9 ± 9.0	24.0 ± 13.9	0.122
Post-op (Day 2)	39.4 ± 26.1	81.2 ± 93.4	**<0.001**	39.9 ± 16.6	46.5 ± 56.2	0.596
Post-op (Day 5)	24.3 ± 11.1	39.7 ± 29.1	**0.046**	25.0 ± 13.2	28.0 ± 22.3	0.567
Post-op (2 weeks)	26.0 ± 17.9	36.8 ± 41.0	0.062	22.1 ± 8.3	25.3 ± 14.5	0.352
Post-op (3 months)	26.8 ± 23.3	32.8 ± 19.8	0.312	25.2 ± 19.8	26.7 ± 16.1	0.745
Post-op (6 months)	25.3 ± 16.8	22.5 ± 7.7	0.492	27.0 ± 20.6	25.4 ± 11.4	0.680
**ALP (U/L)**						
Pre-op	65.8 ± 20.9	66.9 ± 18.4	0.893	59.3 ± 16.0	63.3 ± 16.4	0.351
Post-op (Day 2)	54.2 ± 18.7	62.8 ± 22.1	0.095	55.6 ± 14.1	51.4 ± 13.7	0.248
Post-op (Day 5)	60.0 ± 24.6	71.4 ± 27.2	0.090	55.8 ± 14.6	56.9 ± 20.1	0.825
Post-op (2 weeks)	73.2 ± 24.6	85.4 ± 27.9	0.069	63.4 ± 20.2	71.1 ± 28.1	0.260
Post-op (3 months)	74.0 ± 24.7	78.2 ± 22.3	0.520	71.4 ± 26.5	72.6 ± 23.4	0.851
Post-op (6 months)	77.4 ± 33.3	78.8 ± 19.7	0.865	76.9 ± 21.6	73.1 ± 20.8	0.498
**TB (μmol/L)**						
Pre-op	0.80 ± 0.36	0.98 ± 0.46	0.067	0.78 ± 0.29	0.79 ± 0.32	0.899
Post-op (Day 2)	1.10 ± 0.69	1.12 ± 0.70	0.899	1.01 ± 0.70	1.12 ± 0.83	0.580
Post-op (Day 5)	1.02 ± 0.59	1.24 ± 0.85	0.194	0.96 ± 0.45	1.48 ± 2.72	0.390
Post-op (2 weeks)	0.77 ± 0.50	0.75 ± 0.53	0.922	0.62 ± 0.26	0.72 ± 0.24	0.138
Post-op (3 months)	0.88 ± 0.42	0.90 ± 0.70	0.872	0.87 ± 0.44	0.85 ± 0.35	0.826
Post-op (6 months)	0.91 ± 0.40	1.22 ± 1.13	**0.027**	0.79 ± 0.34	0.94 ± 0.40	0.153

SGOT = Serum glutamic oxaloacetic transaminase, SGPT = Serum glutamic pyruvate transaminase, ALP = Alkaline phosphatase, TB = Total bilirubin. Normal values SGOT = 1–40 U/L, SGPT 1–40 U/L, ALP = 30–115 U/L, TB = 0.2–1.2 umol/L.

**Figure 3:**
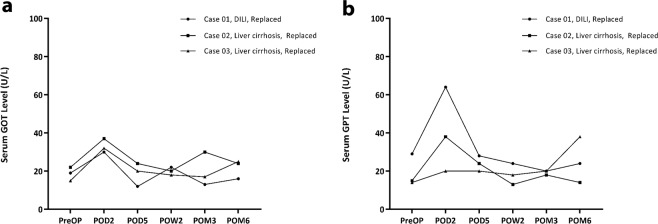
Liver function parameter (SGOT, SGPT) in RLHA ligated patients with preexisting liver disease. There was only transient increased in SGPT level on postoperative Day 2. DILI = Drug-induced liver injury.

In this study, we had 21 patients with pre-existing liver disease. In 15 cases, ALHA was preserved and in 6 cases (3 replaced, 3 accessory), it was ligated. There was only transient increased in SGPT level on postoperative day 2 in RLHA ligated patients (Fig. 3).

### Operative outcome

The operation data and complications are presented in Table 3. The median operation time, blood loss, hospital stay after operation and postoperative complications were not statistically different between the groups. There was no reported liver failure and mortality. Differences on the number of harvested lymph nodes in each station on the lesser curvature side (stations 1, 3, 5, 7, 8a, 9 and 11p) were also not statistically significant as presented in Table 4.

**Table 3:** Operative outcome.

Parameter	Replaced LHA (n = 131)			Accessory LHA (n = 73)		
	Preserved n = 114, (%)	Ligated n = 17, (%)	*P* value	Preserved n = 21, (%)	Ligated n = 52, (%)	*P* value
Operation time	243 ± 73	222 ± 55	0.158	221 ± 59	216 ± 49	0.727
Blood loss	134 ± 126	102 ± 93	0.316	129 ± 126	108 ± 93	0.429
Postoperative stay	9.7 ± 7.5	11.8 ± 8.0	0.295	11.9 ± 9.2	10.9 ± 16.7	0.804
Total complication	16 (14.0)	3 (17.6)	0.713	6 (28.6)	8 (15.4)	0.207
Complication: Grade II or above	14 (12.3)	3 (17.6)	0.463	6 (28.6)	7 (13.5)	0.176

**Table 4:** Number of lymph nodes retrieved in each station on lesser curvature side.

Lymph Node Station	Replaced LHA (n = 131)			Accessary LHA (n = 73)		
	Preserved (n = 114)	Ligated (n = 17)	*P* value	Preserved (n = 21)	Ligated (n = 52)	*P* value
#1	4.3 ± 3.2	4.0 ± 3.2	0.743	4.8 ± 3.2	4.3 ± 3.4	0.601
#3	4.4 ± 4.3	3.1 ± 3.3	0.237	4.2 ± 4.3	5.2 ± 4.4	0.384
#5	0.6 ± 1.2	0.3 ± 0.6	0.054	0.6 ± 1.2	0.7 ± 1.1	0.676
#7	5.5 ± 3.2	5.3 ± 2.7	0.779	4.7 ± 4.1	4.3 ± 3.1	0.630
#8	4.3 ± 3.4	4.7 ± 4.1	0.639	3.9 ± 3.1	3.7 ± 2.9	0.766
#9	3.6 ± 3.0	2.4 ± 3.1	0.129	2.6 ± 2.3	2.9 ± 1.9	0.512
#11p	2.5 ± 2.7	2.9 ± 2.9	0.571	2.4 ± 2.3	1.9 ± 2.1	0.435

## Discussion

Laparoscopic gastrectomy with oncologic lymph node dissection has been recommended for treatment of early gastric cancer in Japan and Korea^16–18^. Evidence have shown that laparoscopic gastrectomy had similar or even better outcome compared to open gastrectomy^19–21^ and is safe even in patients with chronic liver disease^22^. During laparoscopic gastrectomy for gastric cancer, aberrant left hepatic artery is occasionally seen. It is either a replaced artery or an accessory artery. Recent advancement of CT protocol makes preoperative detection and classification of ALHA possible^10^.

Today, there are still lots of debates on whether it is safe to ligate aberrant left hepatic artery during abdominal surgeries. In a study by Okano *et al*.^23^, ligation of aberrant left hepatic artery is safe as it only resulted in transient liver dysfunction. In contrast, other studies have reported severe complications such as abscess formation, cholangitis, liver failure, liver necrosis and even deaths after ligation of ALHA^7–9^. In our institution, we usually preserve the ALHA in early gastric cancer and sacrifice it in advanced cases.

In this study, we classified the ALHA into replaced or accessory type and reviewed each case retrospectively to see if ligation of these arteries during laparoscopic gastrectomy is safe or not in terms of postoperative liver function, surgical and oncologic outcome. Results showed that sacrificing RLHA or acLHA only resulted in transient elevation in SGOT and SGPT levels which spontaneously returned to normal two weeks after surgery. There were no reports of hepatic necrosis even after ligation of ALHA in patients with preexisting liver disease. This phenomenon showed that collateral arterial flow developed after ligation of hepatic artery, which have been demonstrated in the studies of Koehler *et al*.^24^ and Reimann *et al*.^25^

Oki *et al*.^26^ introduced a surgical technique to preserve ALHA but many surgeons believed that preserving the ALHA during laparoscopic gastrectomy will increase the surgical time and amount of blood loss. In our study, there were no significant differences in surgical time, amount of blood loss, number of lymph nodes retrieved in the lesser curvature side, length of hospital stay after surgery and complications among the four groups. These findings shows that ligated ALHA has same postoperative and oncologic outcome as preserving the ALHA.

According to previous reports, elevation of liver enzymes are usually encountered after ligation of large ALHA. We also found the same result in our study. Elevation of serum SGOT and SGPT were seen on postoperative day 2 in the ALHA-ligated group >1.5 mm diameter. Based on the analysis we performed, we recommend that after ligating ALHA >1.5 mm in diameter, liver enzymes should be monitored postoperatively.

A similar study was done by Kim *et al*.^27^ in 2016 but in our study we further categorized ALHA into replaced and accessory type to determine which type of ALHA is safer to preserve or ligate. We also measured the diameter of ALHA instead of LGA as this accurately measures the amount of blood flow to the portion of the liver supplied by the ALHA. This study had several limitations. First, there were only small number of ALHA cases and secondly, it is a single center experience so the total volume of patients enrolled are smaller. Despite of these limitations, we still had sufficient number of patients in each of the four groups that we compared and it showed that ligation of ALHA appeared to be safe as none of the patients suffered adverse outcome. A transient rise in postoperative SGOT and SGPT levels were seen after ligating ALHA >1.5 mm in diameter regardless of subtype. We recommend monitoring liver enzymes postoperatively in these type of patients especially for the RLHA subtype. Our suggestion may also applicable to any upper abdominal surgeries, such as fundoplication, myotomy, hiatal hernia and bariatric surgeries. Since this is a retrospective study, a prospective study involving larger number of patients is recommended for confirmation.

## Data Availability

All data generated or analyzed during this study are included in this published article (and its Supplementary Information files).
